# Neural Networks for Hyperspectral Imaging of Historical Paintings: A Practical Review

**DOI:** 10.3390/s23052419

**Published:** 2023-02-22

**Authors:** Lingxi Liu, Tsveta Miteva, Giovanni Delnevo, Silvia Mirri, Philippe Walter, Laurence de Viguerie, Emeline Pouyet

**Affiliations:** 1 Department of Computer Science and Engineering—Interdepartmental Centre for Industrial ICT Research (CIRI ICT), University of Bologna, 40126 Bologna, Italy; 2 Laboratoire de Chimie Physique—Matière et Rayonnement (LCPMR), UMR 7614, CNRS, Sorbonne Université, 75005 Paris, France; 3 Laboratoire d’Archéologie Moléculaire et Structurale (LAMS), CNRS, Sorbonne Université, 75005 Paris, France

**Keywords:** hyperspectral imaging, neural network, deep learning, cultural heritage

## Abstract

Hyperspectral imaging (HSI) has become widely used in cultural heritage (CH). This very efficient method for artwork analysis is connected with the generation of large amounts of spectral data. The effective processing of such heavy spectral datasets remains an active research area. Along with the firmly established statistical and multivariate analysis methods, neural networks (NNs) represent a promising alternative in the field of CH. Over the last five years, the application of NNs for pigment identification and classification based on HSI datasets has drastically expanded due to the flexibility of the types of data they can process, and their superior ability to extract structures contained in the raw spectral data. This review provides an exhaustive analysis of the literature related to NNs applied for HSI data in the CH field. We outline the existing data processing workflows and propose a comprehensive comparison of the applications and limitations of the various input dataset preparation methods and NN architectures. By leveraging NN strategies in CH, the paper contributes to a wider and more systematic application of this novel data analysis method.

## 1. Introduction

Hyperspectral imaging (HSI) has become widely used in the field of cultural heritage (CH) for painting analyses supporting the identification of original and degraded paint compounds, and revealing underdrawings [1,2,3,4]. The non-invasive and non-destructive analysis tool can be applied in situ in either reflectance, transmittance, or fluorescence mode [5,6,7]. It registers a spatial map and collects a spectrum at each pixel position, in the wavelength range varying from infrared to X-ray. The contiguous set of 2D images collected through the energy range of interest produces a 3D data cube containing millions of spectra, which poses an analytical and computational challenge. Thus, the effective processing of heavy spectral datasets remains an active research area with the primary challenge being to efficiently process data and extract rich information from the inherently complex and delicate painting materials [8,9].

**Figure 1:**
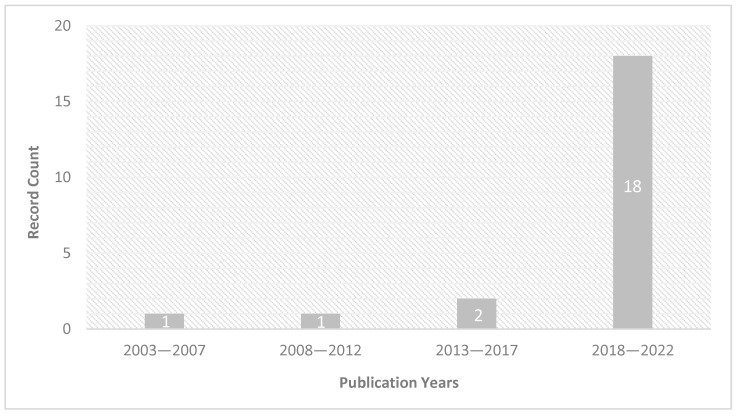
Record count of the scientific publications related to the use of NNs to process hyperspectral datasets from CH paint-based materials over the last twenty years (Web of Science, December 2022 [41], using keywords “paint”, “spectral imaging” and “neural network”).

A range of cutting-edge data processing techniques has particularly been applied in the CH domain to reduce the dimensionality of the dataset, classify and unmix spectral signature to map paint components. Thus, numerous approaches were developed, starting from the conventional multivariate analyses and statistical methods (e.g., spectral angle mapper (SAM) [10,11,12,13], fully constrained least square (FCLS) [14,15,16], principal component analyses (PCA) [17,18,19,20], minimum noise fraction transform (MNF) [21,22], and k-means clustering [23,24,25]), to the more advanced machine learning algorithms (support vector machine (SVM) [26,27], hierarchical clustering [28], embedding techniques [29,30,31,32], MaxD [11,24,33], dictionary learning [34,35]) with a growing interest for neural network algorithms (NNs) [36]. NN-based models first gained a tremendous rise in digital image classification due to their superior ability in feature extraction and pattern recognition [37,38,39,40]. Then, the application of NNs for spectral imaging data drastically expanded over the last 5 years for performing advanced mapping of artistic painting materials (Figure 1) [41]. NNs’ main advantages reside in the extreme flexibility of the types of data they can process, with superior ability to extract hidden and sophisticated structures (both linear and nonlinear) contained in the raw spectral data [42]. This relies on the large flexibility of the NN architectures available (number of neurons, type of layers, and depth), making NNs adaptive for a wide range of tasks and input data [37]. Unlike other machine learning algorithms, the learning of NNs is fully automated, without the need for extensive manual tuning of the underline functions. This powerful and efficient learning ability makes them suitable for complicated tasks and complex painting systems, where the large nonlinear data manifold becomes a merit.

In this circumstance, the rapid rise of NN-based strategies has outlined a variety of data analysis workflows available to the community. However, the flexibility in the architecture designs and the automated learning process (often referred to as “black-box”) cause difficulties in application, and very rare studies have documented their choice on the architecture and appropriately described the use of specific features. In this context, the present article proposes a comprehensive overview of the applications of NNs for HSI of historical paintings. A systematic and exhaustive search was conducted in the time frame up to December 2022 [27,43,44,45,46,47,48,49,50,51,52,53,54,55,56,57,58,59,60,61,62,63,64], which included database searches on Web of Science [41] and manual searches of relevant journals, conference proceedings, and PhD theses. The inclusion criteria were set on implementing NN techniques for processing HSI data in the CH field, with the main focus on solving paint component mapping problems. After defining the basic terminology related to NN in Section 2, we describe the two main applications involving the use of NN for the analyses of data acquired on historical paintings (Section 3). In Section 4 and Section 5 we present adaptations of the dataset and NNs architecture to the specific constraints of ancient and historical materials. Finally, Section 6 discusses and questions the prominent trends that may lead to the future novel implementation of NN algorithms for historical paintings.

## 2. Neural Network Overall Workflow

**Figure 2:**
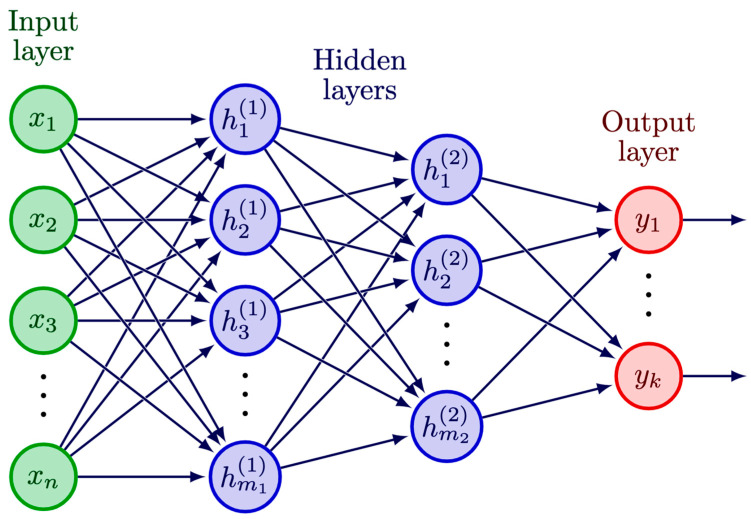
A schematic illustration of an example of a fully connected feed-forward neural network with two hidden layers. *x*, *h*, and *y* represent the neurons in the input, hidden, and output layers, respectively, while *n*, *m*_1_*, m*_2_, and *k* are the total number of neurons in each layer.

NNs are a group of computational algorithms that receive and process information in a way that mimics the neural system of a brain. Most applications in the CH field have adopted the feed-forward structured NN schematically presented in Figure 2 [65]; hence, in the following, we concentrate on this type of NN.

The basic units of a neural network are called “neurons”. Each neuron receives an input, performs a computation, and passes the output to other neurons. The neurons are aggregated in layers of different functionality. The input layer directly receives the input data, its output is passed to the hidden layers which perform the computation, and their output is passed to the output layer which produces the final results. The output of a given neuron is obtained as a weighted sum of the output values of the neurons from the previous layer plus a bias [66,67]. The obtained value is then usually passed through a non-linear activation function, thus producing the neuron output. The ensemble of weights and biases is the model parameters of the NN, and is automatically optimised via learning. A NN with more than one hidden layer is also known as a deep learning (DL) model.

An additional set of parameters, called hyperparameters, controls the speed and the quality of the learning process [68]. These parameters need to be defined before the learning process begins and are further tuned in order to obtain the optimal model for a given dataset. Below we list some of the most common hyperparameters encountered in the CH literature:

- *Number of hidden layers and neurons in each layer.* The number of layers defines the depth of the network and is at the heart of the architecture affecting the performance of a NN [69].
- *Activation functions* define how the inputs to a neuron are transformed into an output to be fed into the next layer, where sigmoid, rectified linear unit (ReLU), and softmax functions are frequently used [70].
- *Loss functions*, such as mean squared error (MSE) and cross-entropy loss, are used to estimate the error between the ground truth and predicted output values [71].
- *Learning rate* defines how quickly a network updates its parameters towards convergence [72].
- *Number of epochs and batch size*. Epochs are the number of times the training data appears to the network and batch size determines after how many input sub-sets the network should update its parameters [72].

In a feed-forward NN, data flows through the network in only one direction, from the input layer to the output layer, without looping back, as in recurrent neural networks (RNNs) [73,74] or generative adversarial networks (GANs) [75,76]. There are different types of hidden layers used according to the specific architecture, such as fully connected layers, convolutional layers, or pooling layers [77,78]. The various NN architectures with different choices of hidden layer types are detailed in Section 5.

After defining the NN architecture and hyperparameters, the datasets are usually divided into a training, validation, and test set. During the training process, the model is presented with a training set, which is a set of samples that the model uses to learn the relationships between the input features and the target outputs. The model parameters (weights and biases) are optimised during this step. The system auto-updates itself through the feeding of input data, without the need to hand-adjust up to millions of parameters in the network. A validation set is used during training to adjust the hyperparameters in order to improve the performance of the model and prevent possible overfitting on the data. In the most common case of feed-forward NNs, the optimisation is accomplished through a process called backpropagation that involves two steps. First, during the forward propagation, the input data is introduced as the input layer, and is transmitted through each hidden layer in sequence until an output is generated. By comparing the output result with the ground truth, an error signal is computed for each neuron in the output layer, indicating the direction and magnitude to adjust. Second, during the backpropagation, the error signals are transmitted backwards from the output layer to each node in the last hidden layer, and repeatedly propagate backwards layer by layer. After the model has been trained, it is tested on a previously unseen test set to evaluate its performance and the ability to generalise to unseen data.

There are several learning strategies that have so far been applied in the field of CH: supervised learning, unsupervised learning, and transfer learning [65,79,80]. In the first case, the available dataset consists of data points and their labels, and the NN algorithm learns a function which maps the input to the output by learning from available labelled examples, as observed in most cases [27,43,44,45,46,47,48,49,53,54,55,56,57,58,60,62,63]. The second type of learning, unsupervised learning, is used to analyse and cluster unlabelled data [51,52,64]. In the case of transfer learning, the model is pre-trained with a large dataset and then fine-tuned with a separate smaller dataset [50,59,61]. This type of learning is discussed in detail in Section 5.3.2.

## 3. NN Application to the Study of Artistic Painting

The ability of NNs to efficiently learn high-level features from vast data has gradually attracted the interest of CH scholars in assisting with spectral data processing.

The preliminary applications of the NN models for CH were implemented on multispectral imaging (MSI) datasets twenty years ago, and focused on the improvement of spectral data resolution and the digital reconstruction of reflectance curves [43,44,45]. Super-resolution spectral reconstruction was first studied in the computer vision field with primary attention on the device-independent digital image reproduction using spectral reflectance as the intermediate space for colour management [17,81]. The very first example of using NNs on multispectral data in CH applications was conducted by Ribés and Schmitt in 2003 [46,47,48], to achieve high fidelity and illumination invariant colour reproduction of fine art paintings. They developed a non-linear model to learn the spectral reflectance curves from multispectral images. Osorio-Gomez et al. later proposed an alternative method for similar tasks on oil canvas paintings [44]. Recently, a similar approach by Shi et al. [43] further optimised faithful print reproduction of oil paintings to model the bidirectional mapping between the spectral reflectance and the 3D printer ink stack layouts. In luminescence MSI development, NNs have also been applied by Chane et al. to learn the radiometrically calibrated emission spectra with resolution in the nanometre scale from 15-band multispectral data [45].

With the significant development and spread of HSI instruments over the last decades, the fast and high-resolution acquisition of the spectral data on artworks has overcome the limitation of multi-spectral sampling. Moreover, in the last five years, the research focus has shifted from spectral super resolution to more practical conservation and restoration related questions. To date, the objectives of implementing NN on the spectral data analysis in CH are mainly focused on paint component identification and the relative spatial visualisation of their distribution. Conventionally, the material identification is performed through the time-consuming interpretations of measured spectral features by CH experts, which is also challenging when associated with complex material mixtures and layered structures. By implementing NN, the pixel-wise identification process could be efficiently achieved using automated data-driven solutions with minimal human intervention. More specifically, the paint component mappings are generally classified into the following categories, further developed below:

1. *Paint component identification* determines the presence or absence of a pigment based on a given spectrum—classification task;
2. *Paint component unmixing* refers to quantitatively decomposing a given spectrum to its base constituents—regression task.

### 3.1. Paint Component Identification

The majority of the research is focused on the first category of tasks that generates a qualitative map of paint components through NN. The simplest case of such application covers the single pigment identification without consideration of pigment mixtures or binding media. Chen et al. developed a model to automatically identify pure pigment areas of heritage paintings based on visible reflectance (VIS-RIS) data in favour of the authentication of artworks [49]. Jones et al. have recently focused on X-ray fluorescence (XRF) data to automatically classify historical pigments used in Renaissance paintings to help understand the artist’s materials and technique [50]. Additionally, a two-step mapping approach has been proposed by Kogou et al. for the material identification of a Peruvian watercolour painting based on macro-XRF data [51]. In this approach, the spectra are first classified into areas with similar features and then the pigments present are hand identified. The proposed strategy has been later applied on visible and near-infrared (VNIR-RIS) data for bronze corrosion products’ identification in a large museum collection [52].

As opposed to the simplified model assumption of a single layer of pure pigments, historical paintings usually feature a more complex mixture of pigments or even have a multi-layered structure. Therefore, a multi-labelling model is necessary for a robust solution to correctly identify the multiple pigments present within every single pixel. Kleynhans et al. have developed a non-linear model to produce a qualitative multi-label map of the intimate pigment mixtures in a single step [53]. Their training dataset is based on VNIR-RIS spectra extracted from well characterised 14th century illuminated manuscripts to make accurate predictions of the materials used by the same art school. In addition to the one-step solution exploiting the hand-labelled database, Grabowski et al. have developed alternative data processing pipelines for a fully automatic pigment classification task [27]. They have broadened the applicability of the algorithms to paintings with unknown material compositions, as they first obtain the labels through automatic identification with several pigment libraries, and then use NNs to classify the entire painting and create a multi-label map.

NNs have also been applied to detect the degradation areas of murals. Mural paintings often suffer from several diseases, such as flaking, deep loss, mud besmirch and sootiness, in various degrees. The documentation of those damaged areas and the identification of their composition is essential for mural conservation and restoration. Sun et al. have applied NN in combination with the dimensionality reduction method, PCA, to identify the degree of flaking in murals at Mogao Grottoes based on spectra in the near-infrared (NIR-RIS) range [54]. Lin et al. adopted a similar approach, combining NN with MNF, to classify the VNIR mural images into various types of damaged regions [55].

### 3.2. Paint Component Unmixing

Several works performed quantitative unmixing of complex paint systems that aim at estimating the concentration of each paint component on a per-pixel basis. Rohani et al. have proposed a quantitative non-linear unmixing strategy of hyperspectral data that involves two steps: first, the NN decomposes the reflectance into its constituent pure pigments members and then the two-constant Kubelka–Munk model is applied to accurately estimate the concentration of pigments in mixtures [56]. The same authors later developed a semi-quantitative pigment unmixing algorithm that relies on training two identical NNs simultaneously [57]. The first NN performs multi-label pigment classification and assigns multiple pigment classes to every spectrum. The second NN tackles the spectral unmixing problem and outputs coefficient maps, thus providing a semi-quantitative mapping of the abundance of the pigment classes. Pouyet et al. adopted the same architecture and applied it to datasets acquired in the short-wave infrared (SWIR-RIS) domain [58]. Another study by Fukumoto et al. applied the encoder–decoder (ENDEC) NN model (fully described in Section 5) that estimates the pigment concentration as an intermediate output and includes a further spectral remix step to reconstruct the predicted spectrum from the estimated concentration [62].

Apart from paint component mapping, NN implementation has also been explored to solve other spectral unmixing problems related to the layered structure of paintings, such as thickness estimation and paint segmentation. Xu et al. have targeted the multi-layered structure of the painting, andapplied a NN to fully automate the pigment identification process [59]. Their work is based on XRF data and has simulated XRF spectra with three-layered pigments to train the model. Shitomi et al. have developed a physics-based ENDEC model (fully described in Section 5), where the decoder part is based on the Kubelka–Munk (KM) model, to estimate the thickness and mixing ratio of pigments [64]. Research conducted by Striova et al. applied the NN to improve the visibility of the pentimenti and underdrawing style based on VNIR multispectral data acquired on a pair of homonymous paintings by Manet and Titian, respectively [60]. Furthermore, in assisting the study of historical painting styles, Zhang et al. have developed a strategy to extract the sketches of damaged or degraded paintings that also exploits spatial features [61]. In a recent advancement, Sun et al. adopted a pre-trained model originally designed for photo restoration to virtually repair the scratched mural paintings that also extend the applicability of NN to digital restoration [63].

## 4. Dataset Preparation

**Figure 3:**
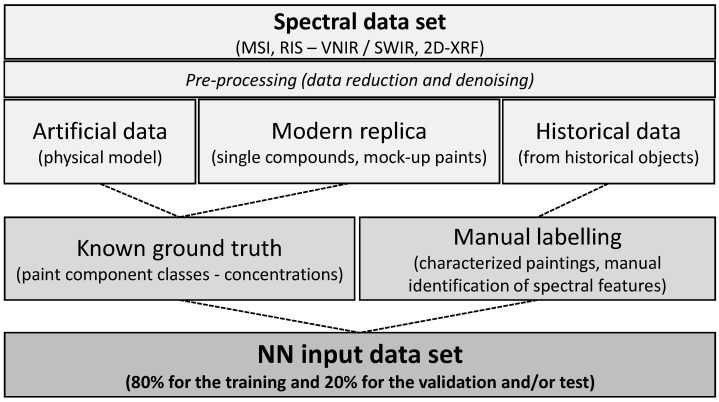
Summary of the dataset preparation workflow.

The spectral input sources, data types, pre-processing steps, and labelling methods are crucial for the dataset preparation. Figure 3 summarises the general workflow across the literature and all the alternatives in this preparation process. After collecting and pre-processing the available data, the obtained dataset is split into two or three subsets: training, validation, and/or test set. A common data splitting ratio is 80% for the training and 20% for the validation and/or test [54,58,60,62]. The alternatives are 64% training, 16% validation, and 20% test according to [56], or 70% training and 30% validation as in [49].

### 4.1. Spectral Inputs

As introduced in Section 3, the early studies were mainly conducted on MSI data for spectral reflectance curves reconstruction. These data were mostly acquired or simulated in reflectance mode, with one exception utilising a 15-band luminescence acquisition in the 450–740 nm range [45]. The reflectance data mainly fall into the VIS domain, either collected with seven narrow-band interferential filters in the range of 480–650 nm [44], or 31 bands with high-dynamic range (HDR) multispectral capture within the spectral range of 420–720 nm [43]. Multispectral data extended to the SWIR range was also inspected, as presented by Striova et al. in 2018, who acquired 32 narrow-band images with spectral coverage from 390 up to 2500 nm [60]. Furthermore, Ribés et al. proposed using artificial data that simulate seven-channel camera responses over the 400–760 nm range [46,48].

In more recent studies, the hyperspectral data were widely used as inputs to the neural networks. The spectral range of the input data varies from RIS to macro-XRF, and the data samples are characterised with improved spectral resolution down to the nanometre scale and increased wavelength channels up to hundreds or even thousands. Some of the studies are based on XRF data, acquired in various experimental conditions (power source, acquisition time, and beam size) [50,51,59]. The RIS data cover several wavelength domains, from the most conventional VIS domain (380–750 nm [62], 400–700 nm [64], 400–720 nm [49]), extended to NIR (383–893 nm [56,57], 377–1033 nm [63], 377–1037 nm [61], 400–950 nm [53], 400–1000 nm [52,55], 822–1719 nm [54]), to the recently emerging SWIR range (930–2500 nm [52], 1000–2500 nm [27,58]).

### 4.2. Data Types and Labelling Methods

#### 4.2.1. Artificial Data

In the shortage of adequately labelled datasets, physical models can be applied to generate a library of synthetic spectra with a sufficient amount of training data. Two approaches have been used in accordance with different spectral inputs: the fundamental parameters (FP) method for XRF spectra and the Kubelka–Munk (KM) theory for RIS data. For these data, the ground truth is known, favouring not only the multi-label classification, but also quantitative estimation or even unmixing in multi-layer structures.

The FP method is based on Sherman’s equations that describe the theoretical relationship between the measured XRF signal intensity of the element and its concentration in the sample [82]. With defined pigments and concentrations, simulated spectra responses are generated. Jones et al. applied this method to create a training dataset containing 3000 spectra of 15 pigment classes [50], modelled as a simplified case that only accounted for the primary fluorescence of single-layered samples. Xu et al. [59] have employed a more complex model that simulates XRF spectra for three-layer structures in mixtures of 12 different pigment classes, resulting in 16,224 spectra in total. Both studies have adopted a transfer learning strategy that further fine-tunes the NN model with a smaller quantity of experimental data, after the initial training with the large synthetic dataset.

For the reflectance data, Kubelka–Munk (KM) theory is widely used to estimate the interaction between the incident light and paint layers [83,84]. This physical model measures a sequence of absorption and scattering coefficients to predict the reflectance spectra from the composition of pigment mixtures. The KM theory can be applied to decompose the measured spectra and thus estimate the pigment concentrations via spectral unmixing. Moreover, it can be used for mixing models that generate many patterns of synthetic spectral data, with given pigment types and concentrations. Rohani et al. have applied a nonlinear KM mixing function to generate simulated spectra of pigment mixtures [56,57]. All possible two/three mixtures out of 11 pure pigments [57] and 12 pigments [56] were modelled and 500 random combinations of coefficients/concentration values were selected for each mixture, obtaining 110,000 spectra and 143,000 spectra in total, respectively. Fukumoto et al. have used a similar approach to build the synthetic dataset based on 19 reference pigments [62]. Two datasets were prepared: the first one that served as a trial to validate the method contained 1771 spectra of only one combination and the second one contained 1,445,136 spectra which included all combinations of 4 colours out of 19, including white with 5% mixing steps. Furthermore, Shitomi et al. synthesized the spectral responses of layered pigments with a given layer thickness and mixing ratio [64]. Three pigments of primary colours are used for the simulation with thickness varying from 0 to 3.2 µm, generating in total 35,700 spectra.

Chen et al. used an alternative method to create an artificial spectral database [49]. They generated augmented pure pigment samples by adding random noise and amplitude offset to the reference pigment database, and synthesised simulated colour mixtures using an ideal subtractive mixing function. All possible mixtures of 3 pigments out of a selected set of 16 pigments were generated with increments of 10%, yielding a total of 21,240 spectra.

#### 4.2.2. Modern Data

Even though artificial data is advantageous regarding the large amount of data and known ground truth samples needed to train a NN, it remains challenging to fully simulate the instrumental and environmental noise, and the real physical processes. To address this issue, many cases included the data acquired on modern samples in the training process. These samples are prepared prior to analysis, including single pigment powders or pellets, and mock-up paintings. The true concentration, or at least the known composition of paint layers, can be used to automatically label the training data.

A few studies have measured the spectral response directly on dry pigment samples as either pigment powders or pellets of a single pigment, with small data sizes up to hundreds of spectra [27,50,52,58]. Most generally, mock-up samples are prepared to imitate the historical painting materiality (binder, pigment(s), preparation layer, and support). The pigments selected to create the mock-ups are commonly based on analysis of the historical artworks and the range of pigment fractions, binder ratios, and layer thicknesses are consistent with those in the historical objects [27,58,59,64]. The spectral data acquired on the mock-ups covered both pure pigment areas and two to three pigment mixtures, with variances in paint thickness in some cases. The input datasets have an average size of 80 k spectra.

In some cases, the mock-ups were used to test the performance of the NNs previously trained on artificial datasets. Rohani et al. created mock-up paintings with selected combinations of mixtures and pure pigment examples to test their model [56,57]. Fukumoto et al. applied their NN on 33 mock-up paintings made by previous work [62], and Xu et al. created a set of mock-up paintings, of which 20% of the obtained spectra were used to fine-tune the model and 80% served as the test case [59].

#### 4.2.3. Historical Data

Historical data are those acquired directly on historical objects. Those data are either included in the training process by forming the input dataset or serving as a test case to verify the performance of the proposed model. The types of art objects and the sizes of the dataset used present a large variability. Within the historical set, both labelled and unlabelled data are used.

Kogou et al. used an unlabelled dataset composed of 41,327 XRF spectra acquired on a Peruvian watercolour painting (c. 1860) by Francisco Pancho Fierro [51]. With the aid of an unsupervised NN model, the large number of XRF spectra was reduced to 13 distinct clusters of unlabelled material compositions. Liggins et al. applied the same model to two excavated bronze fragments, classifying the SWIR and VNIR images with hundreds of thousand spectra (exact size unreported) into three corrosion areas [52]. They also built a reference spectra database composed of 17 samples of powdered and fragmented ancient Chinese bronze extensively characterised by other analytical instruments. The identification was performed by manually comparing the obtained averaged spectra in each cluster with the reference.

On the contrary, supervised networks require labelled input data, which is quite challenging for historical objects that are usually unknown and heterogeneous materials. The main labelling method proposed in the literature relies on the visual inspection of the spectral features, and manual annotation to mark areas with a certain presence of pigments or degradation features. To confirm this manual identification, complementary analyses can be used, such as RIS, XRF, fibre optics reflectance spectroscopy (FORS), X-ray diffraction (XRD), Raman and Fourier Transform InfraRed spectroscopies [27,51,52,54,55]. However, this labelling method is very time-consuming. Thus, usually only small inherent areas with limited pixels are selected, resulting in a much smaller dataset size. Moreover, the obtained labels are not suitable for the quantitative assessment of concentrations.

In some cases, the historical objects in question were extensively studied in previous reports and have been well characterised for all chemical components, available as the ground truth maps. Kleynhans et al. used selected areas on four paintings from the illuminated manuscript Laudario of Sant’Agnese (c. 1340) to form their training dataset [53]. In total, 25 classes of paints were identified, and 16,683 individual spectra were collected across all four paintings. Two out of the four paintings were also used to test the performance of the model. Pouyet et al. also used selected areas of an historical object, a Tibetan thangka dated from the beginning of the 19th century, as part of the training data [58]. They have built their input sets from multiple sources, including single-pigment pellets, pigment mixture mock-ups, and the historical tangka, altogether presenting 12,000 spectra. Striova et al. applied the network based on an oil painting *Madonna of the Rabbit* by É. Manet (c.1856) to improve the visibility of *pentimenti* and underdrawing [60]. 50k randomly selected pixels from the analytical data acquired formed their input dataset.

The above-mentioned data types and labelling methods are mix-used in some studies to build a more reliable training dataset. Both historical data and modern replica data were used to build the input datasets in [52,58]. A large simulated dataset and a smaller mock-up dataset were used in different learning stages in the transfer learning approaches [50,59].

However, historical data are more frequently used for the test of the developed network with the ultimate goal to study the material composition and distribution of historical artworks as illustrated for: one illuminated folio from the 15th century *Book of Hours* in [56]; two impressionistic paintings, the *Poèmes Barbares* by Paul Gauguin (1896) and *The Bathers* by Paul Cezanne (1899–1904) in [59]; a set of 11 paintings by the late Portuguese artist Amadeo de Souza-Cardoso in [49]; and an early Renaissance painting, *Saint Michael Triumphs over the Devil* by Bartolomé Bermejo [50]. In [63], a pre-trained model was tested on damaged murals of a Buddhist temple (c. 1392) to repair the scratches.

### 4.3. Pre-Processing

Several data pre-processing workflows are proposed to improve the data quality before feeding into the NNs. Other than the common calibration process that calibrates the signal with respect to the background and the instrumental environment, the pre-processing mainly focuses on data denoising and data reduction.

First, for RIS data, the low wavelength channels (usually the first 15 bands) or the last bands in the infrared range are usually quite noisy and are removed as noted in [52,54,55,56,57]. For XRF data, taking a spectral range starting from 2.80 keV was suggested in [59] to avoid the peak overlaps in the low energy range that often confuses the model and affects the efficiency. 

Then, the smoothing of the signal is performed by binning and applying various filters to further improve the signal-to-noise ratio. For RIS data, a spectrally moving average filter that reduced the wavelength channels by a factor of 4 was used in [57], and a Savitzky–Golay filter was applied in [49,56] to reduce the high-frequency noise. For the XRF dataset, spatial median filtering with a 3 × 3 pixels kernel and spectral binning of 5 were employed [51]. On the other hand, noise was introduced to the artificial XRF spectra to increase the robustness of the dataset [59].

Data dimensionality reduction techniques, including PCA and MNF, are applied in some cases to centralise the information in fewer bands [44,54,55,63]. An inverse PCA transformation was applied in [54] after processing the first component by high-pass filtering. A pipeline of statistical algorithms, including t-SNE (t-stochastic distributed neighbourhood embedding), DBSCAN (Density-Based Spatial Clustering of Applications with Noise) clustering, HySime (hyperspectral signal subspace identification by minimum error), SISAL (simplex identification via split augmented Lagrangian), and SAM, was built in [27] to fully automate the pigmentidentification process and the results were fed into the NN. Data reduction in relation to balancing the number of samples in each pigment class was also reported in [53]. This task was accomplished by iteratively removing similar spectra in the over-abundance classes (based on measuring Euclidean distance for the similarity) until the samples per class areevenly distributed.

As suggested by Fukumoto et al., selecting candidate pigments can also be considered a pre-processing step [62]. Even though the detail of pigments considered in each study is not listed and compared in this review, it presents a large variability from case to case.

## 5. Model Architectures and Their Evaluation

A wide range of NN architectures has been explored in response to the various research questions, from spectral super-resolution to single or multi-class classification, and from quantitative concentration estimation to spatial feature extraction. In general, as the research problems become more complex, more complicated NNs are implemented to fully learn the structure contained in the input data. In this section, we review the NN models implemented in CH in the time frame from 2003 up to date, roughly in the sequence from the simplest architecture to the more complex ones. We first introduce the unsupervised learning networks with the example of a self-organizing map (Section 5.1), then mainly focus on the supervised approaches, such as multilayer perceptron (Section 5.2) and convolutional neural networks (Section 5.3). In the end, the most complex hybrid approaches are presented, including multi-branches networks that mix-used the above basic types, encoder–decoder networks (Section 5.4), and deep belief networks (Section 5.5). The detailed configurations, structures, choice of hyperparameters, and evaluation methods of the mentioned architectures are summarised.

### 5.1. Self-Organizing MAP (SOM)

A self-organizing map (SOM) is a type of unsupervised learning algorithm, i.e., it does not require a labelled dataset. The algorithm takes the pixel-level spectra as input and maps them onto a 2D layer, the SOM [85]. SOMs thus contain only a single layer of neurons, in which every neuron is associated with a weight vector, with the same length as that of the input vectors. The training takes on the competitive learning strategy, where each neuron competes with its neighbours to be the “winner” for a given input. The winning neuron and its neighbours adjust their weights to better match the input. The SOM NN has a single input parameter—the number of neurons, which should be larger than the number of clusters. This type of clustering method naturally allows for a visualisation of the high-dimensional spectral data in 2D space in the form of single-cluster maps. With each cluster representing a distinct group of similar spectra, the SOM clustering analysis is able to reduce the large number of spectra to a few clusters of similar spectra, thus capturing variations in the density of the paint and the relative concentration of materials [51,52].

SOM has been applied for two different clustering tasks using multispectral XRF data [51], and SWIR and VNIR data [52]. In the former case, the number of final clusters was set to 13. In [52], the authors do not provide information on the number of clusters chosen. In their work, SOM was applied to all spectra from two image cubes. After initial clustering, the number of data points was reduced, and the mean spectrum for each cluster was calculated. A secondary clustering was applied using SOM, grouping the spectra by shape, not by intensity. In order to evaluate the clusters, the quantisation error is computed for each cluster and if it is above a given threshold, the cluster is disintegrated, and the data is subjected to a new round of clustering. The threshold is increased stepwise until all data samples are clustered.

### 5.2. Multilayer Perceptron (MLP)

A multilayer perceptron (MLP) is a fully connected class of feed-forward NNs in which each neuron in one layer is connected to every other neuron in the next layer [86], as illustrated in Figure 2. An MLP typically has three or more layers: an input layer, one or more hidden layers, and an output layer. An MLP with a single hidden layer is considered a shallow NN (Section 5.2.1), while those with two or more hidden layers are regarded as deep learning models (Section 5.2.2) which are frequently denoted as deep MLP or deep neural networks (DNNs). The output layer produces the final prediction, which can be a continuous value (for regression tasks) or a class label (for classification tasks), depending on the activation functions used [70].

#### 5.2.1. Shallow MLP

As an early application, a shallow MLP was applied in [44] to predict reflectance spectral curves. The input datasets are reflectance spectra reconstructed using linear techniques, such as pseudoinverse, PCA, and cubic-spline interpolation. The neural network was composed of an input layer of size 3 × N (where N is the number of channels), a hidden layer of 85 neurons per channel, and an output layer of a single neuron per channel. Linear activation functions were used in the hidden and output layers. The root mean squared error (RMSE) was the metric employed. The neural network parameters were optimised with the aid of the Levenberg–Marquardt algorithm [87,88]. The training was set to stop when RMSE fell below a certain threshold, which was fixed to a small arbitrary value.

A shallow MLP was also applied in [55] to identify mural disease. A three-layer model of 6, 8, and 5 neurons in each layer was developed. The VNIR spectra of 1040 channels were pre-processed by MNF to reduce the spectral dimension and minimise the noise. The first six bands of the transformation results were selected to be the input. The output layer corresponds to the five predicted classes, including diseased and non-diseased areas. A single hidden layer of eight neurons was used. No information on activation and loss function was provided. The number of epochs was set to 1000, the minimum error of the training target was 0.0001, and the learning rate was 0.01.

A shallow MLP classifier was implemented for automatic pigment identification in [27]. They used an off-the-shelf model from the scikit-learn library [89], with hyperparameters set to default values: the model has a single hidden layer of 100 neurons, ReLU activation function, and Adam weight optimizer. The input to the NN are the 256-channel SWIR spectra with pigment labels generated from the previous steps using t-SNE, HySime, SISAL and SAM, and the output returns one of the five pigment classes.

#### 5.2.2. Deep MLP

Pigment identification and spectral unmixing using deep MLP networks were exploited in a series of publications [56,57,58]. In [56], a multi-label DNN with four fully connected layers of 256, 128, 64, and 32 neurons and an output layer of 16 neurons was developed. The sigmoid activation function, whose output is a vector of 0 and 1, was used for all layers. The loss function employed in this model was the average binary cross-entropy loss. In [57] and [58], the authors expanded the first approach from pigment identification to pigment unmixing in the VIS energy range with the aid of a two-branch DNN model.

**Figure 4:**
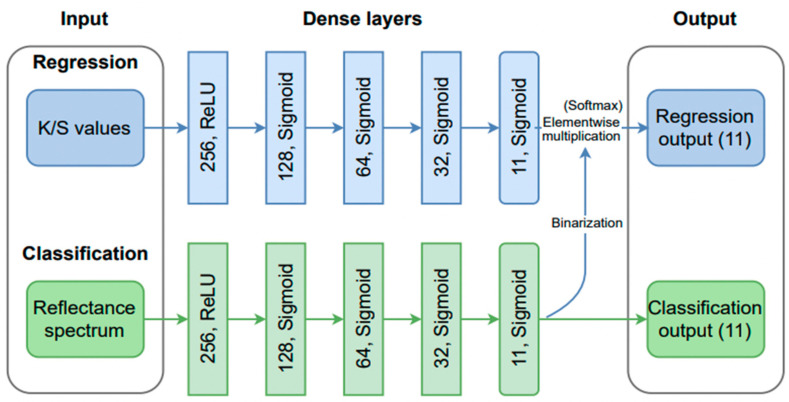
Schematic representation of the two-branch MLP model used in [57,58].

As schematically presented in Figure 4, the first branch of the NN performs pigment identification as a multi-label classification problem as in [56]. The second branch of the DNN model aims at performing spectral unmixing. It is a fully connected network also consisting of four layers of 256, 128, 64, and 32 neurons, with ReLU/sigmoid activation functions. The input layer represents a reflectance spectrum of 256 bands. The output layer contains 11 neurons, whose values correspond to pigment concentrations. To correctly predict the latter, the softmax activation function was employed in the output layer, such that the output values sum up to 1. Adam optimizer with default parameters was used. The batch size was set to 64. The training was performed for 200 epochs. In order to prevent overfitting, early stopping with a patience of 10 was used. The model made use of late fusion, which was shown to give optimal results.

In [60], Striova et al. used a deep MLP composed of five layers to extrapolate NIR reflectance spectra from VIS data. The proposed NN takes visible reflectance spectra from 400 to 750 nm as input and predicts spectra in the NIR range from 1200 to 1700 nm. The input and output spectra both have 16 channels. The neural network is composed of four hidden layers of sizes 15, 15, 15, and 1 neuron(s). The sigmoid activation function was used in each layer, except for the last one where a linear activation function was employed [90]. The model was trained using the scaled conjugate gradient algorithm. The MLP-extrapolated NIR image contains information related to the VIS bands only. Therefore, in order to obtain the pure NIR spectra without the visible contribution, the computed extrapolated spectra were extracted from the measured NIR spectra, and the resulting intensity values were scaled between 0 and 1.

In [43], accurate painting reproduction via 3D printing was achieved by optimising the multi-layer composition of different inks with the aid of a bidirectional model. The model consists of two fully connected neural networks. The first neural network has four hidden layers of 300 neurons each. The hidden layers as well as the final layer use the ReLU activation function. The loss function is the Euclidean distance between the prediction and the measurement (scaled by the square root of the number of wavelength bands/channels). The second neural network takes the 31-band reflectance spectrum as input and predicts the ink layout (an 11-dimensional vector). This neural network consists of eight hidden layers, each layer containing 160 neurons. The ReLU activation function is used for all hidden layers, and a softmax activation function is used in the output layer. This NN is deeper as it attempts to learn a more complicated dependence, namely the prediction of ink layout from reflectance spectra. Two additional loss functions were employed which were shown to produce more stable and consistent training results, namely perceptual loss and thickness loss. Finally, the backward function is trained to minimise the total loss, which is a sum of the spectral, perceptual and layer thickness loss functions. A soft-quantisation layer is applied at the end of the layout prediction layer in order to force the neural network to predict close-to-integer values for the number of ink layers.

### 5.3. Convolutional Neural Networks (CNNs)

A convolutional neural network (CNN) is a specialised type of deep neural networks that uses convolution operation in at least one of the hidden layers to down-sample the input data and extract features. A convolutional layer is made up of a set of kernels (the set is also known as a filter) which are matrices of weights, optimised during training. The input is convolved with the filters to produce a feature map. In the case of CNNs the neurons are sparsely connected, as opposed to the full connectivity in MLPs, which means each neuron only receives input from a restricted area around it. Frequently, one or more convolutional layers followed by a pooling layer form a convolutional block. There are usually multiple convolutional blocks followed by a fully connected block to produce the final outputs. Different types of blocks can be connected in various ways to form a customised architecture for the designed task [91,92].

#### 5.3.1. One-Dimensional-CNN

One-dimensional (1D)-CNN is a CNN that processes one-dimensional input data. Input data for CNNs are often two-dimensional, i.e., an image, taking advantage of CNNs’ capacity to learn the spatial correlations. However, in the CH literature, spectral input is commonly used, i.e., series of spectra serve as the inputs to the network without maintaining the spatial relationship.

**Figure 5:**
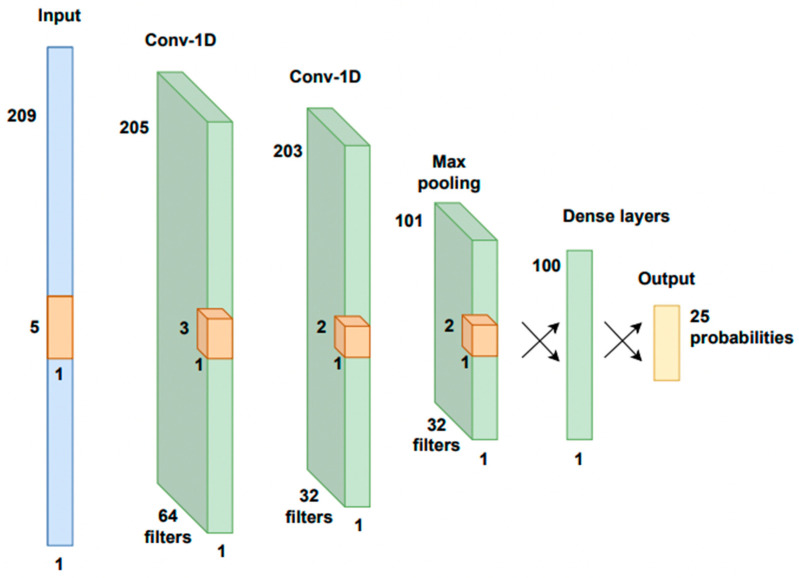
Schematic 1D-CNN network architecture used in [53]. The input layer (shown in blue) takes as input the VNIR spectrum, followed by a series of hidden layers (shown in green) that perform feature extraction using convolutional kernels (shown in orange). The output layer (shown in yellow) produces the final classification results.

Kleynhans et al. trained a typical 1D-CNN model for multi-class pigment classification based on VNIR data [53]. As summarised in Figure 5, the proposed model consists of one input layer, four hidden layers, and one output layer. The input layer has one dimension with 209 neurons, which is the wavelength steps number of the input spectra. The first two hidden layers are 1D convolutional layers with, respectively 64 and 32 filters and kernel sizes of 5 × 5 and 3 × 3. This is followed by a max pooling layer, which down-samples the previous hidden layer by 2 to reduce the dimensionality, keeping the maximum value of every second neuron. Then, two fully connected layers of sizes 100 and 25 form the last two layers. The output consists of 25 classes corresponding to the labels of the input spectra, including both pure pigments and pigment mixtures. The ReLU function is used as activation function for each hidden layer, while softmax is used for the final layer, which gives a probability value between 0 and 1 for each class, summing up to 1. The final class is assigned by defining a threshold; at 0.99, a number of pixels remain unclassified. By decreasing the threshold from 0.99 to 0.85, part of the unclassified areas was assigned a class with rising in false positive identifications.

The performance of the model was measured with a categorical cross-entropy loss function, while the stochastic gradient descent optimizer was applied to minimise the loss function. For the learning process, the model was trained with batch sizes of 50 and epochs of 30, and evaluated on a validation set of 10% of the training data. The training started with a learning rate of 0.01, which was decreased if after four epochs (cycle through full training dataset) the validation loss did not decrease.

#### 5.3.2. CNN with Transfer Learning

Transfer learning (TL) is a learning strategy in which a model trained on one set of data is reused as the starting point to train a new model on data with slightly different properties. Both models adopt the same architecture but possess different parameters. The low-level features learned by the pre-trained model are maintained when training a new model by freezing all parameters on certain layers, commonly the earliest layers which tend to learn more general structures from the input set. The model, thus, only adjusts a smaller portion of parameters to adapt to the new data, making it possible to achieve high accuracy with much less training data and computational resources. TL is especially useful when the available data is limited while the NN has a rather complex architecture, as the model can leverage the knowledge gained from the larger dataset to the new task. In CH, two works have adopted the TL strategy to exploit large artificial datasets [50,59].

In [50], a CNN model was applied to classify XRF spectra into pigment classes (15 classes were used in this study). The network was composed of three convolutional layers with max-pooling layers in between, and the output layer predicts the label for the corresponding pigment. The ReLU activation function was used for each layer except the final layer, which used the softmax activation function. The model is pre-trained on synthetic XRF data, and then fine-tuned with spectra acquired on real pigment samples, by freezing the weights in all layers in the network except the final layer. The performance of the model was evaluated before and after the fine-tuning, and the model with transfer learning showed higher accuracy.

A more complex CNN model was developed in [59] to identify layered pigments. The inputs were XRF spectra with 3815 channels. The model consists of five convolutional blocks, each made up of a 1D convolutional layer, a batch normalisation layer, and a max-pooling layer. The number and size of the kernels of each 1D convolutional layer were set at 64, 64, 64, 64, 128 and 5, 3, 3, 3, 3, respectively. The model is then followed by a post-convolutional layer with 128 kernels with a size of 3, a flatten layer, a dropout layer and a fully connected layer. The activation function LeakyReLU is used for every layer other than the final layer, while the output layer uses a sigmoid activation function. The output predictions were in 11 classes, outputting the probabilities between 0 to 1 for each pigment class. The loss was calculated to optimise the performance of the model by averaging the binary cross entropy of each predicted class.

The model was pre-trained with the artificial dataset using randomly initialised weights. It was further fine-tuned with data acquired on mock-ups, in two training steps: first, the pre-trained weights were fixed in all convolutional layers, whereas only the fully connected layers were fine-tuned. Next, all layers were trainable and were further fine-tuned with the mock-up dataset.

#### 5.3.3. Multi-Branches CNN

Two different types of complex CNN architectures have so far been employed to analyse hyperspectral data from painted artworks: the first one to perform sketch extraction [61], and the second one to identify pure pigments [49].

In the first case, an efficient edge-detection algorithm, consisting of a combination of two NNs, a bi-directional cascade network (BDCN) [93] and a U-net [94], was employed. BDCN consists of five incremental detection (ID) blocks, which contain convolutional layers and a scale enhancement module (SEM). Each block is connected to the next one by a pooling layer. A carefully designed loss function making use of a distinction between edge and non-edge pixels was employed. To account for the imbalanced distribution of edge and non-edge pixels, a class-balanced cross-entropy loss was employed [61]. Due to insufficient training data, the authors resorted to transfer learning by pre-training the BDCN model on a publicly available dataset of natural scenes. The network parameters were then fine-tuned on a target cultural relics dataset.

In [49], Chen et al. achieved pure pigment identification with a multi-class classification problem using a three-branch deep-learning (DL) model. Unlike the 1D-CNN where a single spectrum is used as input, in this case, the first two branches of the NN take as input a small sub-cube around a central pixel, thus allowing for a combined spectral and spatial investigation. The first branch of the neural network consists of five sets of 3D convolutional filters with ReLU activation functions. It takes a 9 × 9 sub-cube of the hyperspectral cube around a central pixel as input. The architecture of the second branch is identical to that of the first one using the derivative of the same sub-cube as input.

The third branch of the model takes the reflectance spectrum of the central pixel as input and computes the spectral correlation map between the pixel and a reference pigment database. This result is fed to a shallow fully connected feed-forward NN, which analyses the error between the reflectance spectrum of the pixel and the spectral signatures in the reference pigment database.

The outputs of the three branches are flattened, concatenated in a single vector, and fed into a fully connected feed-forward deep NN of five hidden layers with ReLU activation functions. One of these layers is a dropout layer, intended to minimise overfitting. The output layer consists of 17 neurons—16 for the pure pigments, and 1 neuron which predicts whether the reflectance spectrum belongs to a mixture or not. A threshold of 0.9 is applied to the output to discriminate between the spectra of pure pigments and pigment mixtures. The categorical cross-entropy loss function was employed. The NN parameters were optimised with the aid of the Adam optimizer with a learning rate of 0.0001. The training was performed for 20 epochs.

### 5.4. Encoder–Decoder (ENDEC)

**Figure 6:**
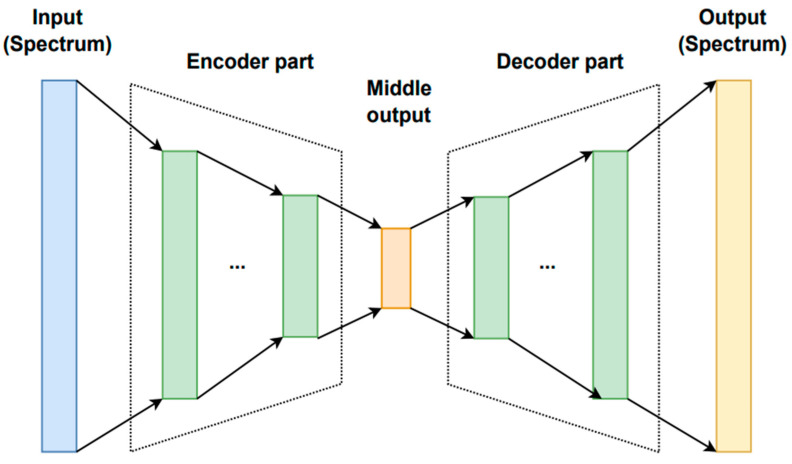
Typical architecture of an encoder–decoder neural network. The input (shown in blue) is first compressed by the encoder part (shown in green) into a lower-dimensional representation (shown in orange). The decoder part (also marked in green) then performs up-sampling from the middle output and produces a reconstructed version of the input (shown in yellow).

An encoder–decoder (ENDEC) is a neural network model which consists of two parts—an encoder and a decoder. The two parts usually have inversed structures, with the encoder working in the normal direction that compresses the input data into a lower-dimensional representation (latent), and the decoder reconstructing the input from the latent, thus working in the opposite direction [95,96]. The typical structure of an ENDEC model is illustrated in Figure 6. The hidden layers can be either fully connected, convolutional or recurrent, based on the specific architecture [97]. The encoder and decoder are trained together by minimising the difference between the reconstructed outputs and the inputs. The training can be performed through either a supervised or unsupervised learning strategy as the ground truth is already provided in the input data; thus, in some cases, no explicit labels are needed.

A fully connected ENDEC was used in [62] in order to learn the dependence of reflectance spectra on pigment concentrations. The input and output of the ENDEC are reflectance spectra, whereas the intermediate layer represents pigment concentrations. The encoder part consists of eight layers of 300 neurons each, and an output layer of 19 neurons (for 19 pigments). The ReLU activation function was used in all layers, except for the output layer, where the softmax activation function was employed. The mean absolute error (MAE) between the ground truth and the predicted concentrations was the loss function in the encoder part. The decoder part is composed of four hidden layers of 500 neurons each; the ReLU activation function was used for all layers. The mean squared error was the loss function of the decoder part. The Adam optimisation algorithm with default parameters was employed. In order to avoid overfitting, L_2_ regularisation was applied, with a weight decay value of 1 × 10^−5^. The learning was first performed only in the decoder unit, then the decoder weights were fixed, and the encoder part was trained.

Another type of encoder–decoder architecture, based on CNN and known as U-net [94], was used in [61] to refine and denoise sketches extracted by a BDCN. The encoder NN consisted of four blocks each composed of three convolutional layers and a pooling layer. The decoder part of U-net consisted of four blocks composed of three convolutional layers. Weight decay regularisation was employed to reduce overfitting.

An unsupervised autoencoder model is proposed in [64] for paint layer thickness and pigments mixing ratio estimation. It is a type of ENDEC network that utilises unlabelled data for training, with the goal to learn a compact representation of the input data (middle output). Since the ground truth of the reconstructed spectrum is the input data itself, the model is also considered self-supervised. In this case, the input of the encoder is spectral data of layered surface objects and is decomposed into latent variables in the middle layer. The decoder part is based on the Kubelka–Munk theory; as such, the latent variables are physically interpretable as pigment thickness and pigment mixing ratio. The encoder consists of seven fully connected layers with ReLU activation function, and the number of neurons in each layer is 300. For the middle output layer, a ReLU function is used to force the thickness to be positive and a softmax function is used for pigment mixing ratios. A special loss function is designed, and the Adam algorithm is used for optimisation. The training was repeated in three cases with different types of inputs: artificial data, data measured on mock-ups of tomb mural pigments, and that of watercolour pigments.

A variation autoencoder (VAE)-based network was applied in [63] for recovering large areas of scratch damage in mural paintings. The model adopted is pre-trained in a previous study [98] designed for old photo restoration; thus, no training process is involved and the murals serve as test cases to the pre-trained model. Since the VAEs are able to learn compact representations of input data (encoder part) and generate new data following the probabilistic distribution (the decoder part is also known as the generator), the model is capable of repairing the lost contents in murals that suffer from similar damages of photos. The input to the NN is a true colour image synthesised from VNIR datacube, which is pre-processed and enhanced to assist the restoration. The model consists of two identical VAEs and an adversarial mapping network (GAN). The two VAEs take the damaged images and the related ground truth images as inputs, respectively, where the encoder transforms the input into a compressed representation in latent space (middle output), and the decoder part generates a reconstructed version of the input image. Then, the middle outputs of the first VAE (damaged images) are translated into that of the repaired ones of the second VAE through the mapping network. The encoder part and decoder part are both composed of three convolutional/deconvolutional layers and four residual blocks, and the mapping network has six convolutional layers, six residual blocks, and one partial non-local block.

### 5.5. Deep Belief Network (DBN)

Deep belief networks (DBNs) are a type of NN that consist of multiple layers. In a DBN, the lower layers consist of the so-called restricted Boltzmann machines [99,100], typically used for unsupervised learning and designed to learn the underlying probability distribution of a dataset, and the top layer is usually fully connected. The training process of DBN is different from MLP and CNN, which involves pre-training the lower layers using unsupervised learning and then fine-tuning the top layers using supervised learning through backpropagation [101]. DBN can be used to solve unsupervised learning tasks to reduce the dimensionality of features, as well as for supervised learning tasks to build classification or regression models.

In [54], a DBN-initialised neural network was used to predict the degree of flaking. The NN is composed of two hidden layers, with the number of neurons configured as 200 and 150, respectively. The weights of the DBNs are pre-trained layer-by-layer, from the first hidden layer to the outermost layer; the learning rate was set to 0.01 for 100 epochs, and the momentum was set to 0.4. No information on activation functions and output layer is provided.

### 5.6. Model Performance Evaluation

The testing of the generated output on an unseen dataset is accompanied by different evaluation criteria. For the test sets, the ground truth is already known by the methods described in Section 4.2 and the quantitative evaluation of the prediction accuracy is performed [27,53,54,55,56,59,61,62]. As summarised in Table 1, it is measured by calculating sensitivity, overall accuracy (OA), average accuracy (AA), standard deviation (STD), R-squared coefficient of determination, root mean square error (RMSE), or using the individual per-class accuracy. For those tested on a new set of historical paintings, the performance is evaluated more qualitatively. The output results of the networks are commonly cross-validated with other analytical instruments, involving the manual check of spectral features of selected points using XRF, FORS, FTIR or micro-Raman, and optical microscopy [49,50,51,56,58,60].

The performance of the NNs is also frequently compared with other methods and algorithms. In [53], it is compared with the conventional two-step approaches that first perform a spectral clustering and then label the present pigments based on additional information. In addition, the performance of the NN models are particularly compared with other linear unmixing algorithms, including support vector machine (SVM), spectral angle mapper (SAM), and fully constrained least squares (FCLS) [49,53,56,58]. In [53], the 1D-CNN is proven to outperform the SAM by 2.1% and the SVM by 6.4%, respectively, in identification accuracy. In [58], the DNN classification model outperformed SAM by 5.1% in accuracy for pure pigments, with the latter performing poorly in identifying pigments present in mixtures or from areas where the endmembers are not priorly defined. FCLS is tested in [56] for decomposing the given spectrum to a linear combination of pure pigment spectra; however, the accuracy of estimated mixing coefficients largely underperformed the DNN model proposed. SAM and FCLS are also tested in [49] for identifying pure pigment areas by setting a threshold, while the performances are far from the multi-branch CNN model developed.

In some cases, the performances of different NN architectures are also evaluated and compared. The CNN-based model is compared with the unsupervised SOM in [61], the 1D-CNN model is compared with MLP and outperforms the latter in [53], and the unsupervised autoencoder is tested to be superior to the supervised model in [64]. Moreover, in developing the algorithms, NN models with different algorithmic choices are tested and evaluated. The DBN is compared with an artificial NN-based approach in [54]; two MLPs performing multi-class and multi-label classification, respectively, are compared in [56]; and four DNN architectures with different fusion stages are tested in [57].

## 6. Discussion and Conclusions

The promising adoption of NNs within CH is evident throughout the literature. The NN models that learn and adapt on their own are relatively easy to build and, once the model is properly trained, the application to real problems is very fast, i.e., it takes only tens of seconds.

As summarised in Table 2, we have demonstrated the wide applications of NNs in solving paint component mapping problems, from pure pigments to complex mixtures, from classification to concentration estimation, and from multi-class to multi-layer systems. Different architectures were chosen according to multiple factors, from fully connected networks to the more advanced convolutional networks or encoder–decoders, from shallow single-layer networks to complex multi-branch NN models, combined with unsupervised learning to supervised or transfer learning strategies. Furthermore, the training datasets also present a large complexity, varying in spectral ranges, input data types, and preparation methods, from artificial data to historical data, and from modern model paintings to historical artworks of different origins.

Over the last few years, various algorithms were developed with the aim of capturing complex features from HSI data for an in-depth analysis of the rich spectral features [102]. However, as observed in the remote sensing field, the scale of the HSI training data is limited due to the cost, complexity, and labelling constraints in their development [103]. That, in turn, causes a sub-optimal learning of NN with large numbers of parameters. The complexity of the architecture and the variability of the data is often unbalanced, which frequently causes poor applicability to real-world problems. In this circumstance, a specific discussion, concerning the datasets and the NNs architectures, is addressed, for a more systematic use of neural network algorithms for hyperspectral imaging in CH.

Supervised NN models represent a major part of the models developed for classification or unmixing purposes (85.7%). However, due to the costly and labour-intense labelling of HSI data, the supervised models suffer from very limited labelled input datasets that constrain the effective learning of the massive spectral parameters required for accurate classification or regression results [104,105]. Extensive use of those models will soon represent a bottleneck that will significantly inhibit their practical application for wider HSI data analysis. Building new HSI benchmark datasets to meet the training needs of the NN model is expensive and time-consuming [106]. Several HSI datasets have been assembled over the last few years for diverse applications, and multiple open-source databases of diffuse reflectance reference spectra flourished simultaneously [107,108,109]. However, the currently labelled HSI samples are still far below the demands of the current NN models. Limited training samples cause model overfitting during training which significantly affects the model performance. Overfitting can be desirable when the application is specific to a set of data that is mostly similar to the training set, as the model can make very accurate predictions. However, in most cases, it is good practice to avoid overfitting to increase the applicability of the model and make it more resistant to noise and other types of variations.The generation of artificial training data, simulated through theoretical approximations, has been proposed to produce new training samples to mitigate the cost of preparing multiple modern replicas [49,50,56,57,59,62,64]. The available training data size is largely expanded: the averaged data sizes for modern replicas and historical data are around 80 k and 12 k, respectively, while the artificial sets generated have on average 250 k spectra (Table 2). Similarly, several works address this issue by expanding the dataset through an augmented dataset approach [49], and a mixed use of various dataset types (artificial, modern, historical) [50,52,58,59].

In CH, a general trend has been observed with NN algorithms becoming more powerful and widely applied, leading to increasingly complex architectures to tackle more difficult tasks. This trend is driven by the increasing complexity of data and the improvement in computing power. However, it is important to balance the complexity of the network with the size of the dataset in order to achieve the best performance and efficiency. It is generally recommended to start with a simple network configuration and gradually increase complexity to find the optimal architecture for a given dataset, and to reach the optimal model performance in terms of accuracy and computing power. For large and complex datasets, a more advanced network with more hidden layers and complex structures is preferred, while a simpler network is sufficient for smaller datasets and simpler tasks. This practice is observed in the CH literature where relatively simpler models (SOM and MLP) have an averaged training set size of around 80 k spectra, while for more complex architectures (CNN, ENDEC and DBN), the average size is 215 k spectra (Table 2).As an example, [50,59] adopted a CNN architecture and transfer learning strategy, respectively, while using the same input range and data type. As the model used in [59] is much more complex than in [50], the size of the training data is also significantly increased. 

## 7. Perspectives

In conclusion, spectral imaging instruments have become a common tool in CH studies accompanied with a natural extension of dataset and data range. The use of neural networks (NNs) is likely to become increasingly important for assisting the interpretation and information extraction of hyperspectral dataset. As such, the approach will soon grow and provide more examples, available to the community. In this context potential research axes arise from this review. Many researchers have indeed recognized the limitation of current practices in terms of poor generalisation ability and applicability to other datasets, highlighting the need for new, larger and more complex reference hyperspectral datasets. One way to address this issue is to continue to increase the dataset by including a wider range of data from larger pigment databases and more variable painting structures. In order to facilitate this process and increase model transferability, a move towards open-source algorithms and publicly available datasets is indispensable. Similarly, the combination of NN models with complex physical models (four-flux KM approximation [110,111], or the radiation transfer equation [111,112]) needs to be explored, providing more diverse and accurate artificial data to feed NN models. In parallel to data augmentation and synthetic data generation, thatstarted to be addressed in the field, NN models that can efficiently utilise a limited number of labelled samples have to be tested. As such, transfer learning or weakly supervised methods should be further explored to mitigate the demand for training samples [113].

## Figures and Tables

**Table 1:** Most commonly encountered metrics for the quantitative evaluation of prediction accuracy in CH.

Metrics Type	Formula	Ref.
Sensitivity	$\mathrm{Sensitivity}=\frac{True\;positive}{True\;positive+False\;negative}$	[59]
Overall accuracy (OA)	$OA=\frac{\#\;of\;correctly\;classified\;examples}{Total\;\#\;of\;examples}$	
Average accuracy (AA)	$AA=\frac{1}{M}\overset{m}{\underset{i=1}{\sum }}OA_{i}$, M—number of classes, OA_i_—overall per class accuracy	[27,53,55,59]
Standard deviation (STD)	$STD=\sqrt{\;\frac{1}{N}\overset{N}{\underset{i=1}{\sum }}{\left(x_{i}-\mu \right)}^{2}}$, N—total # of examples, *𝛍*$—\mathrm{mean}\;\mu =\frac{1}{N}\overset{N}{\underset{j=1}{\sum }}x_{j}$	[27]
Coefficient of determination ($R^{2}$)	$R^{2}=1-\frac{RSS}{TSS}$ $RSS=\overset{}{\underset{i}{\sum }}{\left(y_{i}-y_{i}^{pred}\right)}^{2}$—sum of squared residuals $TSS=\overset{}{\underset{i}{\sum }}{\left(y_{i}-\bar{y}\right)}^{2}$—total sum of squares, $\bar{y}$ – mean value	[54]
Root mean square error (RMSE)	$RMSE=\sqrt{\;\frac{\sum_{i=1}^{N}{\left(y_{i}-y_{i}^{pred}\right)}^{2}}{N}}$	[54,56,61,62]

**Table 2:** A summary of the neural network models analysed in this review. The distinct characteristics of the NN models are presented, such as the spectral input utilised, the type and size of the dataset employed, the assigned task, and their applications to historical objects.

Architecture	Spectral Input	Input Type *	Dataset Size (Spectra)	Task	Application	Ref.
SOM	XRF	III	41,327	Clustering	Peruvian watercolour painting (c.1860)	[51]
	RIS (SWIR)	II, III	NA	Clustering	Excavated bronze fragments (B.C.)	[52]
MLP	RIS (VNIR)	III	NA	Classification	Mural paintings (17th–20th century)	[55]
	RIS (VNIR)	I	143,000	Classification	II, illuminated folio (15th century)	[56]
	RIS (VNIR)	I	110,000	Unmixing	II	[57]
	RIS (SWIR)	II, III	12,000	Unmixing	Tibetan thangka (19th century)	[58]
	RIS (SWIR)	II	NA	Classification	II	[27]
CNN	XRF	I	16,224	Unmixing	II, impressionistic paintings (c.1900)	[59]
		II	1320			
	XRF	I	3000	Classification	Early Renaissance painting (1468)	[50]
		II	75			
	RIS (VIS)	I	21,240	Classification	Late Portuguese paintings (c.1910)	[49]
	RIS (VNIR)	III	16,683	Classification	Illuminated manuscript (c.1340)	[53]
ENDEC	RIS (VIS)	I	1,445,136	Unmixing	II	[62]
	RIS (VIS) **	I	35,700	Unmixing	II	[64]
		II	213,000		Decorated tomb murals	
		II	171,600		Watercolours	
	RIS (VNIR)	III	NA ***	Restoration	Mural paintings (c.1392)	[63]
CNN, ENDEC	RIS (VNIR)	III ****	500 public images and 41 relics images	Edge detection	Painted cultural relics	[61]
DBN	RIS (NIR)	III	12,000	Classification	Mural paintings (7th–9th century)	[54]

* Input type I, II, and III refers to artificial data, modern data, and historical data described in Section 4.2. ** The three different input sets serve as separate training cases in ref. [64]. *** Ref. [63] used a pre-trained model developed by a previous study; thus, the application does not include the training process. **** Ref. [61] used a publicly available dataset containing 500 images to pre-train their model, which is the only case and does not belong to our defined categories.

## Data Availability

Not applicable.
